# Miscellaneous Notices

**Published:** 1855-04

**Authors:** 


### MISCELLANEOUS NOTICES.

*Baltimore College of Dental Surgery.*—The fifteenth annual commencement of the Baltimore College of Dental Surgery was held in the Assembly Rooms of the College, on the evening of the 1st of March. The exercises were opened with prayer by the Rev. Dr. Johns; after which was read, by the Dean, the following list of graduates, with their subjects of theses:

| | |
|---|---|
| Samuel Belford, Pa., | Exostosis. |
| De Witt Clinton Benbow, N. C., | Carving. |
| John Henry Bond, Md., | Inflammation of the Mouth and parts adjacent. |
| George Jacob Conner, B. A., Pa., | Ether and Chloroform. |
| Joshua Caleb Curry, Ga., | Preservation of the Temporary Teeth. |
| George Richard Hy. Duff, Ky., | Arsenic. |
| Ferdinand J. S. Gorgas, B. A., Pa., | Dentistry as a Profession. |
| Hugh McGinnis Grant, Va., | Dental Caries and its Treatment. |
| Chapin Bond Harris, Md., | Salivary Glands and the Saliva. |
| West Harris, N. C., | Insertion of Pivot Teeth. |
| Randal Duke Hay, M. D., N. C., | Refining Gold. |
| Benjamin Dorr Hyde, Md., | Glandular System. |
| John Jones, N. C., | Disease of the Maxillary Sinus. |
| James Warner Kilpatrick, N. C., | Caries of the Teeth. |
| Christenberry Lee, S. C., | Irregularity of the Teeth. |
| Anderson Roscoe Miller, N. C., | Evacuants. |
| George Washington Pelletier, N. C., | Dental Caries. |
| Frederick Henry Rehwinkel, Ohio, | Causes of Dental Caries. |
| Addison Exum Ricks, N. C., | Sympathy. |
| Rufus Scott, B. A., N. C., | Anæsthesia. |
| William Shakespeare Tate, N. C., | Structure and Diseases of Teeth. |
| Theodoro Suzzara Verdi, Italy, | Eruption of the Dentes Sapientiæ. |
| James Thomas Walton, Va., | Relation of Anatomy and Physiology to Dentistry. |
| John Henry Wayt, M. D., Va., | Labium Leporinum in connection with Fissure of the Palate. |
| Wm. G. Westmoreland, M. D., Ala., | Constitutional Effect of Diseased Teeth and Gums. |
| Joseph White Wiley, Pa., | Cancrum Oris. |
| Adoniram Judson Wright, N. Y., | Mechanical Dentistry. |

The Graduates then received their Diplomas from the President, and were afterwads addressed by Dr. Wm. H. Dwinelle, of Cazenovia, N. Y. This address, which our readers will find in the present No. of the Journal, demands and will richly reward something more than a hasty perusal—the usual fate of valedictories. The most careless reader cannot fail to be roused by its eloquence; while to the heart of the thoughtful, and the high minded it will speak cheering and encouraging words in their efforts to raise our art above the degrading influences of ignorance, unskillfulness and quackery.

*The Alumni of American Dental Colleges.*—The Third Annual Meeting of this Society was held in the Chemical Hall of the Baltimore College of

Dental Surgery. The President, Dr. W. W. H. Thackston, having been prevented from attending by sickness in his family, the meeting was called to order by Dr. C. W. Ballard, first Vice-president. The opening Address was delivered by Professor A. A. Blandy. The reports of committees and papers of gentlemen appointed at the preceding meeting were ordered to be published. Ten or fifteen persons were admitted to membership.

The officers for the ensuing year are—

Professor Robert Arthur, President.
Dr. C. W. Ballard, First Vice-president.
" W. H. Dwinelle, Second Vice-president.
Professor A. A. Blandy, Corresponding and Recording Secretary.
" P. H. Austen, Treasurer.

The Society adjourned to meet in Philadelphia on the day of the next Annual Commencement of the Dental College of that city.

*Experimental Physiology.*—It is with great satisfaction that we announce to our readers the establishment of a professorship of this important branch of medical science in the University of Maryland. So much attention has been recently bestowed upon this subject in Europe, that it is inexcusable in us to neglect it. The advantages of such a course, and the general appreciation of its merits by students of medicine in America, have been sufficiently established by the success of M. Brown Sequard, of Paris, in the northern cities of our Union. We have no doubt that the students of our own city will show the same interest in this eminently useful study.

We consider the University extremely fortunate in securing the service of a gentleman so eminently qualified for the post as Dr. Christopher Johnston. In addition to excellent natural abilities, he has enjoyed peculiar advantages for the prosecution of these studies, and has availed himself of them with the most praiseworthy industry. A considerable time spent with the most distinguished physiologists of Europe, and an untiring study of the phenomena of nature at home, have fully qualified him for the duties of the responsible position to which he has been called.

*Illustrations of Crystalline Gold.*—We had the pleasure of listening to some remarks from Dr. Dwinelle on the subject of crystalline gold, delivered before a few of the dentists of Baltimore, during his recent visit to our city. He gave a history of the gradual progress of discovery in the preparation of this important article and described the instruments necessary to be used and the manner of employing them.

His remarks gave great satisfaction to the gentlemen present, who went away with a higher opinion and a better understanding of the crystalline gold than they had previously had.

***Philadelphia College of Dental Surgery.***—The third Annual Commencement of this institution was held Wednesday evening, February 28th; on which occasion the degree of Doctor of Dental Surgery was, we believe, conferred on fifteen of the class. The valedictory was delivered by Professor Robert Arthur.

***The Dental Register.***—We have received from the author, Dr. G. H. Waters, of Waterbury, Connecticut, the above named work. It is designed to keep a minute record of all dental operations, and so admirably is it arranged for this purpose, that it serves both as a day book and leger, while the particular part of every tooth operated on is indicated, and may be referred to at any future period. Every other page is furnished with diagrams of the teeth of both jaws arranged in straight lines, running from left to right. The particular operation may be marked upon the teeth operated on, so that all the posting that is required is, to carry out the amount to the right. The operations on twelve months may be recorded on every two pages. The most superficial examination of it will be sufficient to convince any one of its value. This, or a similar book, should be used by every dentist.

***Auditory Apparatus of the Mosquito.***—Dr. C. Johnston, of our city, has published an article in the last number of the London Microscopical Journal on this subject. The paper is handsomely illustrated with accurate representations of the facts described.

The paper shows that there is a capsule on the inner margin of each lunated eye, into which the antenna of that side is implanted. The capsule is double, the inner layer receiving the antenna, and the outer receding from the inner one, at every point except the base, so as to leave a space between the two layers. This space is the *auditory capsule.*

It is filled with an opalescent fluid of moderate consistency, containing minute spherical granules. The antennar nerve, coming from the first or cerebral ganglion, after entering the pedicle of the double capsule, divides into two sets of filaments, the central threads of which pass on into the antenna, while the peripheral ones radiate outwards into the capsular space, where they are lodged in sulci wrought in the inner wall of the capsule.

The whorls of stiff hairs, which surround each joint of the antennæ, receive the atmospheric vibrations, while the antennæ themselves transmit these vibrations to the fluid of the capsule. "The animal may judge of the *intensity* or *distance* of the source of sound by the *quantity* of the impression; of the *pitch* or *quality,* by the consonance of particular whorls of the stiff hairs, according to their lengths; and of the *direction* in which the modulations travel, by the manner in which they strike upon the *antennæ,* or may be made to meet in either *antenna,* in consequence of an opposite movement of that part."

It was our intention to have given a fuller abstract of this paper, but we have been very much pressed for room in the present number.

*Crystallized Gold.*—The use of this preparation, as a substitute for gold foil, has, during the last one or two years, attracted much attention, and elicited considerable discussion; and although the use of it has been decried by a few members of the dental profession, its advocates nevertheless are rapidly multiplying. The senior editor has, up to this time, used about eight ounces, made by A. J. Watts & Co., and thus far his expectations with regard to its value have been fully realized. He does not believe, however, that it will ever wholly supersede the use of foil, but that it is superior to it for many operations, he is equally convinced. Its plasticity, and the ease with which the crystals may be welded and made into a perfectly solid mass, enables the dentist to secure more perfectly the preservation of some teeth, than can be done by the most skillful manipulation with foil, and, when necessary, to build up and reproduce the crown of a tooth when lost. His observations and experience also warrant the belief, that a filling of crystal gold, properly made, can never crumble or give way, and the adhesion of it to the surrounding walls of dentine is such as utterly to preclude the admission of moisture.

The great interest manifested by the profession in the use of this new preparation of gold, has induced us to give place to a lengthy and ably written article upon the subject, by Dr. W. H. Dwinelle. It is the fullest exposition of its properties and the method of using it, of anything which has appeared upon the subject. It will be read by our subscribers with pleasure; but in giving place to it, we are compelled to exclude not only our quarterly summary, but also most of the selected articles designed for publication in the present number, and to curtail very greatly our editorial limits. The great value of the article in question, however, is such, that our readers, we feel assured, will pardon the omissions.

*Porcelain Teeth*—We have a few specimens of porcelain teeth made by the New York Manufacturing Company, which, for compactness of structure and strength, beauty of shape, and resemblance to the natural organs in color and semi-translucency, are equal, we think, to any we have ever seen. Among the samples in our possession are some designed to be mounted with continuous gum, and better calculated to secure strength in cases where they can be employed, than any we have seen. We have also some specimens manufactured by Messrs. Oram & Armstrong, of Philadelphia, which resemble more closely, than any we have seen, those made by S. W. Stockton about twelve or fourteen years ago, and which, for resemblance to the natural teeth and strength, have not since, in our opinion, been equalled.

DR. HARRIS:

*Dear Sir*—I most cheerfully avail myself of your kind permission extended to me this evening to jot down a few paragraphs for the forthcoming Journal. I cannot conceive how I can better promote the interests of your readers and the cause of truth, than by correcting several errors—of the head, I am confident, and not of the heart—which I find in an article written by Dr. A. M. Leslie, D. D. S., for the current number of your Quarterly, entitled a "Report on Dental Progress," designed, I have no doubt, to give a just exposition of the advancement of our profession, but which unfortunately is calculated, in many important particulars, to misrepresent the actual condition of dental progress, and also to do great injustice to some of those to whom the profession are under peculiar obligations.

Dr. Leslie is mistaken in assuming that England was the first to make a preparation of sponge gold. Dr. C. T. Jackson, of Boston, discovered a method by which gold could be produced in this form. Although the sponge gold of Dr. Jackson did not prove a practical material for our profession, yet he is fully entitled to the credit of taking the initiative in this direction. The preparation of Dr. Jackson occurs in an amorphous condition—indeed, it most resembles powder held together by a slight affinity. The English gold of Barling, though structural, is not crystal-formed. Dr. A. J. Watts' gold alone is crystalline.

Dr. A. J. Watts' interests have materially suffered from the similarity existing between his name and that of Dr. Watt, of Xenia, Ohio—Dr. Leslie's co-laborer upon the committee. Dr. Watt, of Ohio, seemingly, is not satisfied with appropriating to himself Dr. A. J. Watts' property in his patent, but needs must appropriate his name also. Dr. Leslie, in his manuscript, falls into the error of writing Dr. Watt's name *Watts*, and so it is printed in the Journal. If Dr. Watt is honest in this, he must have been re-christened since the publication of Dr. A. J. Watts' patent and specifications in the Dental Recorder of July, 1853. For some time past the profession have been kept in a state of lively expectation, ready to bestow their gratitude upon their prospective benefactors, Drs. Taft and Watt, whenever they should be favored with the promised discovery of their method of making crystal gold. Up to this time, however, their willing ovation has been unwillingly withheld in consequence of the very judicious silence of the "discoverers."

We are placed in a position of painful perplexity by a remarkable and most lucid instalment of the promised information given in the article of Dr. Leslie, by which we learn that Drs. Taft and Watt have "intimated that they are indebted to Turner and other chemists for the *groundwork* of their process!" We are perplexed to know how to properly bestow our gratitude—whether to reserve its full expression till the final denouement, or to imitate our benefactors in dealing it out in fractions!

Drs. Taft and Watt are indebted to Turner and other chemists for the groundwork of their process, just in the way that all poets, historians, and authors of every sort, are indebted to a standard dictionary for the groundwork of their productions, and not otherwise. The process by which Drs. Taft and Watt produce their practical article of crystal gold has no special groundwork in Turner's or any other chemistry as yet published. They have derived their whole system of operation from the published specification of

A. J. Watts, of Utica, who was the first to discover the method of making all varieties of crystalline gold now in use by the profession, and for this he obtained his patent. The statement of Drs. Taft and Watt, that they have made any discoveries in regard to the crystallization of gold, is entirely without truth. Every article which they have produced and brought before the public is identical, *in all respects,* with those made by A. J. Watts, of Utica, as far back as April, 1853, and they are all produced by precisely the same means that Watts employed and prescribed in his patent, in whose broad field of detail and specification, Taft and Watt, and White, of Utica, have "discovered" all their "discoveries."

Dr. Taft betrays a degree of unblushing effrontery and consummate coolness truly astonishing, when in speaking of his method of manufacturing crystal gold he tells us that he prefers crystal gold made after Watts' patent, as published in the Dental Recorder of July, 1853. The inference is plain, that he willingly admits that all of the profit and advantage which he derives from the manufacture of crystal gold is gained by appropriating the property of another to his own use, which is absolutely the case. It is not often that crime assumes such an impudent and boastful phase, and petty larceny generally has the modesty at least to attempt to conceal its shameless depravity; but it seems there are exceptions even to this rule.

The fact is, that neither Taft nor Watt have anything new to communicate, and this is the whole secret of their backwardness in giving the world their promised information, and also explains why Dr. Leslie derives so little information from Dr. Watt, his co-laborer on the committee.

Dr. Taft is mistaken in his statement in regard to the manner in which gold crystals are formed by amalgamation with mercury. The crystals are originally formed crystals of amalgam, and the subsequent treatment only drives off the mercury, leaving the gold in the crystalline form which the amalgam assumed. As to electric action, the notion is absurd.

After several unsuccessful attempts to gain an interest in Watts' patent, White, of Utica, wrote to Washington for a copy of Watts' specifications, and while waiting for them, boasted of his ability to get around the patent. Shortly after, we find him in the market with an article of gold, not only similar in appearance, but actually admitted by him to be made by a modification of Watts' patent.

At the time Watts obtained his patent he had covered generally the whole ground of his discovery, and found himself capable of producing at will more than an hundred varieties, each product being as fixed and definite in its character as the combinations which produced it.

The article produced by precipitating a solution of gold by protosulphate of iron did not originate with Dr. Taft, nor can it be made of practical value to the profession.

Dr. Leslie takes issue with me when I assert, that "gold cannot always be made chemically pure by quartation, for the reason that gold often contains foreign metals not acted upon by acids." The word acid was misprinted

"acids;" this explanation, taken in connection with the term "quartation," will be sufficient, I think, to show that I referred to the familiar and usual process of purifying gold by nitric acid. I think my friend, the Dr., will know how to make due allowance for typographical errors, if I may judge from the large number which have crept into his article. There are several other and superior methods of purifying gold than by "cupellation" or "cementation." The article explaining the method to which I particularly referred shall be forthcoming.

Quartation will not always render gold pure, for the reason that native gold often contains platinum, palladium, rhodium, iridium, iridosmin, ruthenium, and other metals not acted upon by nitric acid. Even silver itself is not completely extracted by this process, even though the operation is repeated several times, and conducted with great care. A small trace of silver in a large mass of gold will be sufficient to change its color perceptibly from that of pure gold.

I most emphatically endorse the statement of Dr. Leslie, that "for want of chemical knowledge, great ignorance prevails respecting the purity of gold foil." Dr. Leslie will find much important information on the subject of gold in Piggot's Dental Chemistry, pages 289 to 312, inclusive. He will there find many of his erroneous impressions corrected, not only in reference to the character of gold, but also as regards its manufacture into foil. Dr. Leslie is mistaken when he thinks gold-beaters do not alloy their foil. Many manufacturers practice it constantly, and justify themselves on the ground, that it is better. The fact that some of the most popular foil manufacturers furnish articles of two and even three qualities, I supposed was familiar to every one. Both copper and silver are used in combination with gold for the purpose of producing their varying qualities.

I have no doubt but that many manufacturers use every effort to render their gold absolutely pure, but the color of their foil often proves that they have not been successful, for reasons which I have given above.

I spoke of pure gold being of a uniform color throughout the world, and referred to Abbey's foil and some others as representing the color of the pure in contrast with the paler and less pure foils of the market. Abbey's foil is not always absolutely pure, but I believe it is generally very nearly so, and for this reason I referred to it for comparison. A number of years ago, Abbey's foil was quite red, owing probably to some peculiar treatment in its manufacture. It is not so now, its present hue is a fair representative of the color of pure gold. If a few leaves of it are melted into a button, and this is afterwards burnished, it will present the same appearance with an equal amount of burnished foil.

The color of pure crystal gold varies with its texture and crystalline form, in consequence of the differing angles at which light is reflected from its surface, but when condensed and burnished, its tint is ever the same.

On page 236, twelfth line from the bottom, Dr. Leslie probably intended to quote me as recommending the operator to *stipple* and not to *"slope"* the surface of the stopping. This is fully treated in an article in the present No. of the Journal.

The remaining part of the report on Dental Progress will form the subject of an article in the next number.

The experience of Dr. J. D. White, of Philadelphia, with crystal gold, seems to have been a very remarkable one, if we may judge from various articles published in the Dental News Letter during the past year. In the April No., 1854, of that Journal, in speaking of crystal or sponge gold, he says of it, "some specimens when they are damp will not work well, and others would seem to work better with water!" It is not remarkable that gold, when in the form of foil or sponge, does *not* work well under water, but that it should work *better* under water is truly astonishing.

The doctor's experience with crystal gold seems to be entirely the reverse of that of others. He succeeds where others fail, and fails where they succeed; under extraordinary circumstances he is peculiarly successful, but in legitimate practice with crystal gold he fails. In all of my own operations, I am remarkably unsuccessful in this kind of *sub-marine* practice.

The doctor seems to have a singular *penchant* for mingling water with his gold fillings of all kinds. In the July News Letter, 1854, he recommends that a sort of hydropathic treatment be constantly observed in filling teeth. He tells us that "*dampness* must permeate a plug to some extent, or the dampness will force around the plug and displace it sooner or later!"

I can understand that the doctor considers dampness an essential element of a good plug, but I cannot appreciate the force of his *reasoning*.(?) He virtually says, if dampness wont, dampness will—if dampness cant, dampness can. He warns us against making plugs too hard for the reason that they cannot be permeated by the fluids of the mouth! he objects to crystal gold plugs because they are so hard, that dampness cannot permeate them! indeed the doctor occupies all sides of the question. In one place he tells us that crystal gold "can be applied in some cases very well"—in another, he does not think a permanent filling can *ever* be made with it. He objects to the hardness of crystal gold fillings, and then complains of their softness. He assures us that "dampness of the mouth on the tooth will break up the *texture* of the plug, and cause it to crumble out" and then again if dampness *does* permeate a plug, "the dampness will force *around* the plug and displace it sooner or later."! If this is reasoning, it is certainly reasoning in a circle.

The doctor says, "no reasonably good operator loses a plug by softening, but by the margins of *his* cavity giving way." It seems to me that this very softening of the gold is the reason why the margins do give way; of course the acrid fluids which they absorb and retain cannot act upon the gold—the dentine and enamel are their natural prey. Any foil or other filling that is permeated by the fluids of the mouth fails to answer the purpose for which it is designed; it becomes an offensive receptacle of vicious and acrid secretions, which being then retained as within a sponge, insidiously and most rapidly result in the sure destruction of the tooth. W. H. DWINELLE.

